# The potential for effective reasoning guides children’s preference for small group discussion over crowdsourcing

**DOI:** 10.1038/s41598-021-04680-z

**Published:** 2022-01-24

**Authors:** Emory Richardson, Frank C. Keil

**Affiliations:** Department of Psychology, Yale University, 2 Hillhouse Ave, New Haven, CT 06520-8205 USA

**Keywords:** Psychology, Human behaviour

## Abstract

Communication between social learners can make a group collectively “wiser” than any individual, but conformist tendencies can also distort collective judgment. We asked whether intuitions about when communication is likely to improve or distort collective judgment could allow social learners to take advantage of the benefits of communication while minimizing the risks. In three experiments (n = 360), 7- to 10-year old children and adults decided whether to refer a question to a small group for discussion or “crowdsource” independent judgments from individual advisors. For problems affording the kind of ‘demonstrative’ reasoning that allows a group member to reliably correct errors made by even a majority, all ages preferred to consult the discussion group, even compared to a crowd ten times as large—consistent with past research suggesting that discussion groups regularly outperform even their best members for reasoning problems. In contrast, we observed a consistent developmental shift towards crowdsourcing independent judgments when reasoning by itself was insufficient to conclusively answer a question. Results suggest sophisticated intuitions about the nature of social influence and collective intelligence may guide our social learning strategies from early in development.

## Introduction

When is advice from multiple people more likely to clarify than confound a learner’s understanding? Consider two ways one could learn from multiple people at once: by eliciting a consensus judgment from a small group discussion, or by “crowdsourcing” many independent answers. Discussion may enable groups to correct mistakes and combine insights, producing an accurate consensus answer that no individual could have found alone. However, without an objective method of evaluating solutions, discussion may drag on endlessly, be misled by charismatic leaders or groupthink, and ultimately only create an illusion of consensus around a wrong answer. In contrast, crowdsourcing may still include mistakes that discussion could have corrected, but particularly in *large* crowds of *independent* responders, the majority, plurality, and average response can all be surprisingly accurate^1,2^. Nevertheless, shared culture and cognitive biases can create illusions of consensus even without direct communication between individuals^2–4^. The empirical advantages of discussion and various crowdsourcing strategies are well-documented. Less attention has been given to laypeople’s own intuitions about the tradeoffs between them (note that our use of “intuition” does not refer to the intuitive-deliberative distinction in dual systems theory; rather, it follows the frequent use of “intuitive theories” in developmental psychology to describe the untaught assumptions about the world that help learners structure their experience; for a recent review, see Gerstenberg & Tenenbaum, 2017^5^). Here, we investigate early developing intuitions about when group discussion or crowdsourcing is a more effective use of collective intelligence.

While debate over whether crowds can be trusted is at least as old as philosophy itself^6,7^, mathematical models suggest that under certain conditions, crowds can be “wise”. Given a set of options to “vote” for, majority and plurality accuracy increase to near certainty as crowd size increases^8,9^, and evolutionary simulations suggest that conformist learning strategies are often more adaptive than alternatives^1,10^. Similarly, averaging crowd members’ individual judgments can produce a collective estimate that is more accurate than the crowd’s most accurate member^2,11–14^. Interestingly, many species faced with the problem of learning from multiple sources at once rely on similar heuristics to evaluate collective opinion. Even in early childhood, people trust majority over minority judgment, and give more weight to stronger majorities^15^. By adulthood, people also trust pluralities, and give more weight to the judgments of larger crowds^16–18^.

However, crowdsourcing heuristics share a common weakness: for crowds to be “wise”, individual judgments must be independent. Social influence can compound individual error, particularly when a large proportion of the population are conformist learners^19,20^. Yet, while popular concerns about echo chambers and media bias suggest that laypeople intuitively recognize some of the risks of social influences, it remains unclear how well people compensate for them in practice. For example, while adults and even children as young as six prefer firsthand knowledge over hearsay in an eyewitness memory context^3,21^, adults are just as trusting of an economic forecast repeated by five news articles citing a single primary source as they are of the same forecast citing five different primary sources^3,22^. Similarly, while children as young as four expect randomly sampled evidence to cause others to revise their beliefs more than evidence from a biased sampling process^23^, when the source of the sampling bias is *the selection of informants itself*, children are sometimes insensitive to bias even late in development—particularly when the degree of consensus is high^24–29^. Indeed, even as adults, people frequently mistake the frequency of a belief in their local networks for its frequency in the population as a whole^30–32^. In short, people’s trust and distrust of consensus seems to selectively disregard one of the proposed preconditions of consensus’ accuracy—independent sources.

One reason for people’s occasional indifference to their sources’ independence may be that social influence frequently makes their judgments *more* accurate^33–38^. For instance, while open discussion may risk groupthink by sacrificing individuals’ independence, it also allows individuals to pool their knowledge and generate new insights; discussion can also ease the cognitive load on individuals, increase a groups’ capacity for processing information, and allow the group to correct individual mistakes^39–41^. This division of cognitive labor means that discussion groups may be able to quickly generate solutions that most individuals would never produce alone, and may make discussion an attractive learning strategy for a wide variety of problems, particularly as the evidence load increases^42–45^. Most notably, to the extent that discussion enables even a single group member to correct a *majority* that has made a mistake, discussion may also have an advantage over crowdsourcing heuristics like majority rule^46^. Studies of group problem-solving have suggested that this ‘truth wins’ effect occurs when a shared conceptual system enables individuals to conclusively *demonstrate* that a given answer is correct or incorrect—and it is the strength of their argument, rather than the individual’s confidence or simply the presentation of the correct answer, which predicts whether the majority will be persuaded. Importantly, these studies suggest that “demonstrability” is a matter of degree, ranging from mathematics as the “preeminent domain of demonstrability” to purely judgmental tasks such as attitudes and preferences, with a variety of evidence-based reasoning and insight problems also being high in “demonstrability”^47–49^. Note the implication of the “truth wins” effect for social learners: if a minority is able to demonstrate that their judgment is accurate, the majority is not simply *influenced* by the judgment of the minority, they will *defer* to it. Thus, when demonstrations are possible, discussion groups may offer substantially more accurate collective judgments than a “crowdsourced” majority, with little risk of distorting an accurate majority judgment. Indeed, recent accounts suggest that reasoning itself may be most naturally deployed in service of argumentation and function most effectively in interpersonal contexts^50,51^.

Note that we are not claiming that group discussion is *only* beneficial for questions that afford demonstrative reasoning, or that demonstration and reasoning are synonymous. Rather, we focus on discussion and crowdsourcing as flexible, commonsense approaches to a fundamental problem for any social learner: integrating information from multiple sources without inheriting their errors. Our intent is to examine laypeople’s intuitions about their tradeoffs. Though crowdsourcing heuristics like majority rule can be remarkably accurate, they also presuppose independent judges—an unrealistic assumption about human societies. Meanwhile, work on group problem-solving has repeatedly found that discussion not only allows groups to outperform heuristics like majority rule, but that their ability to do so depends on the “demonstrability” of the problem^49–57^. Of course, demonstration is possible without reasoning (e.g., by physically demonstrating how an artifact works or showing the location of an object), and reasoning cannot always conclusively demonstrate a that a solution is optimal. However, reasoning may be a reliable means of correcting errors even when physical demonstrations are not feasible, and when a correct answer cannot be simply deduced. For example, knowing the distance from New York to Chicago won’t allow a group to deduce the distance from New York to Cleveland, but it may enable them correct some over- and underestimates without needing to actually measure the distance. Indeed, in a recent comparison of group discussion with the wisdom of crowds on a numerical estimation task, the average collective estimate of four small-group discussions was more accurate than the average of 1400 individual estimates, and participants reported arriving at their estimates by “sharing arguments and reasoning together”^53^. In short, to the extent that people expect to be able to rely on demonstrative reasoning to minimize the risks groupthink, it may be intuitive to disregard the importance of independent judgment, even if they favor crowdsourcing heuristics in other cases.

In the present work, we asked whether people would favor different social learning strategies for problems that afford demonstrative reasoning than those that do not. Crowdsourcing independent judgments may be more valuable when the potential for reasoning is less salient, particularly when the crowd is large. Discussion may be more valuable when demonstrative reasoning provides a reliable means of analyzing problems and identifying errors, even if the discussion group is small. Because past work suggests that sophisticated social learning strategies emerge in early childhood but also that children appear to underestimate some risks of social influence even in late childhood^21,26^, we focused on adults and children ages 7–10. Understanding how the ability to balance the risks and benefits of social influence develops could shed light on the incongruence of our remarkable capacity for collective problem-solving and our apparent susceptibility to groupthink. It may also provide clues as to where interventions to thwart misinformation may be most effective.

**Figure 1:**
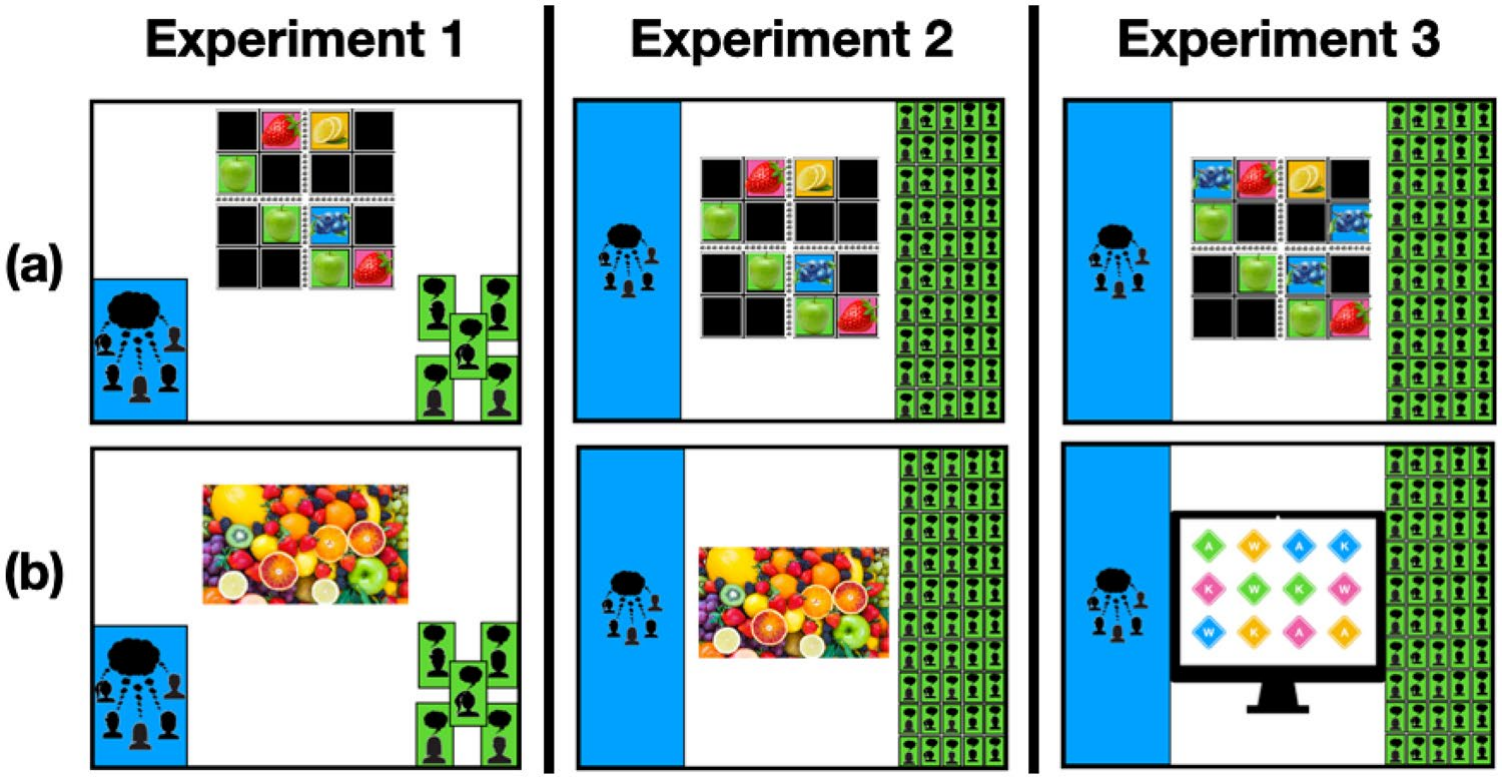
Example stimuli: Groups and Crowds. Each participant saw 8 questions. Experiments 1 and 2 used the same questions (4 *Reasoning*, 4 *Population Preference*). Experiment 3 contrasted *Easy* versions of the *Reasoning* questions with *Hard Perceptual Discrimination* questions. (**a**) Top Row: Example *Reasoning* question. Two-by-two Fruit Sudoku from Experiments 1 & 2 & partially completed “Easy” version in Experiment 3. (**b**) Bottom Row: Example *Population Preference* question (most popular fruit in the world) and *Perceptual Discrimination* question (which shape is spinning the fastest).

In each experiment (Fig. 1), participants were shown eight questions (4 *Reasoning* and 4 *Non-Reasoning*), and for each question, they rated whether crowdsourcing or discussion would be more helpful in answering, on a 4-point scale. In Experiment 1, this meant that participants rated whether it would be more helpful to ask five people to each answer independently, or to ask the same five people to give a single group answer after discussing. In Experiments 2 and 3, we contrasted the five-person group discussion with a crowd of 50 people answering alone. For the *Reasoning* questions, we chose a set of constraint-satisfaction problems that would challenge adults’ capacities, but still be understandable to children (e.g., Sudoku). Because the solutions to these questions must satisfy a mutually understood set of explicit constraints, discussion can help groups generate potential solutions and and reduce processing demands on individuals while relying on demonstrative reasoning to correct errors. In Experiments 1 and 2, we contrasted the *Reasoning* questions with *Population Preference* questions (e.g., most popular fruit in the world). Though individuals’ intuitions may sway as the discussion generates potential answers, discussion provides no objective means of adjudicating disagreement; thus, it may distort intuitions rather than sharpen them. In Experiment 3, we contrasted easy versions of the same *Reasoning* questions with a set of challenging *Perceptual Discrimination* questions (e.g., fastest rotating item in an array), which a separate sample had rated as more difficult than the *Reasoning* questions. This allowed us to test the role of perceived difficulty against the potential for effective reasoning. If participants simply favor discussion for questions that feel more difficult—regardless of whether discussion can reliably adjudicate disagreement—then the preference for group discussion will be stronger for the *Perceptual Discrimination* questions than the *Reasoning* questions. Our general prediction in all three experiments was that sensitivity to the contrast between reasoning and intuitive judgment would lead all ages to prefer group discussion for reasoning questions. However, because past work has suggested that children may underestimate the risks of social influence until between the ages of 6 and 9^21,23,24,26^, we predicted that a robust preference for crowdsourcing non-reasoning questions would emerge only among older children (ages 9–10) and adults, while younger children (ages 7–8) would favor group discussion for both kinds of questions in Experiments 1 and 3. All experiments were preregistered, and the data, materials, and power analyses (https://osf.io/6pw5n/) are available on the OSF repository. All experiments were approved by the Yale University Institutional Review Board and conducted according to their guidelines. Written informed consent was obtained from all adult participants. Because children participated online, parents were recorded reading the informed consent form aloud.

## Experiment 1

### Method

#### Participants

 We recruited 40 adults through MTurk, as well as 80 children (40 Younger, M = 8.01, SD = 0.56; 40 Older, M = 9.92, SD = 0.56; 39 girls). Children participated through an online platform for developmental research that allows researchers to video chat with families using pictures and videos on slides^54^. Sample size was chosen based on the estimated effect size from pilot results.

#### Materials

 We asked eight test questions (Fig. 1), four from each of two question types: *Reasoning* and *Popularity*. Questions were presented from the perspective of a protagonist (Jack). The *Reasoning* questions were chosen to be simple enough to explain to children, but challenging enough that the answer would not be immediately obvious to adults. (1) A 4 × 4 Sudoku puzzle adapted for children. (2) A vehicle routing problem that required a MarioKart character to find the shortest road through all the treasures on a map without taking “two in a row that are the same color, or two in a row that are the same shape”. (3) A single-heap game of Nim (“Each side takes turns picking up pencils. Each turn, you can pick up either 1, 2, or 3 pencils. The winner is the person who picks up the last pencil. There are 5 pencils left in this game; how many pencils should Jack pick up?”). (4) An “impossible object” puzzle that requires the solver to remove a dowel held in place by a nut and bolt from inside a bottle without breaking the dowel or the bottle. The *Popularity* questions concerned the most common subjective preferences in a population. (1) Whether pizza or hot dogs were more preferred by students in Jack’s school. (2) What most people in the world say their favorite fruit is. (3) What most people in the world say their favorite day is. (4) What most people at Jack’s school say their favourite color is. Questions were written to have approximately equal word counts (M_Reas_ = 73.75, M_Pop_ = 67.75). Three counterbalances were created to vary the order of the questions—Forward, Reverse, and a Shuffle. Color coding of answer choice and left/right presentation were also counterbalanced between participants.

#### Procedure

 Children were introduced to the protagonist, Jack (a silhouette). They were told that Jack was unsure of the answers to the questions, and could ask five people for help. The five people could either help by *Talking Together* (giving Jack a single answer as a group), or by *Answering Alone* (each giving Jack their own answer after thinking about the question without consulting others). For each item, children and adults rated whether “talking together” or “answering alone” was “probably more helpful, or definitely more helpful”, producing a 4-point scale of relative preference, where 1 corresponds to “definitely answering alone”, and 4 corresponds to “definitely talking together.” Adults used the scale directly; children’s responses were staggered: they first chose the more helpful strategy, and then were asked for a “probably/definitely” judgment. After answering the eight test items, participants were asked the two comprehension check questions (these were not counterbalanced: Comp_TT was always presented first). Two features of the procedure are important to keep in mind. First, participants could not evaluate the content of any answer to any question, because none was given: they were asked to choose a *means* of advice, not evaluate the quality of the advice itself. Secondly, they could not make judgments based on *degree* or *quality* of consensus—they only knew that the group would have to give one answer, while the crowd would have to give 5 independent answers which could differ or not.

#### Results

**Figure 2:**
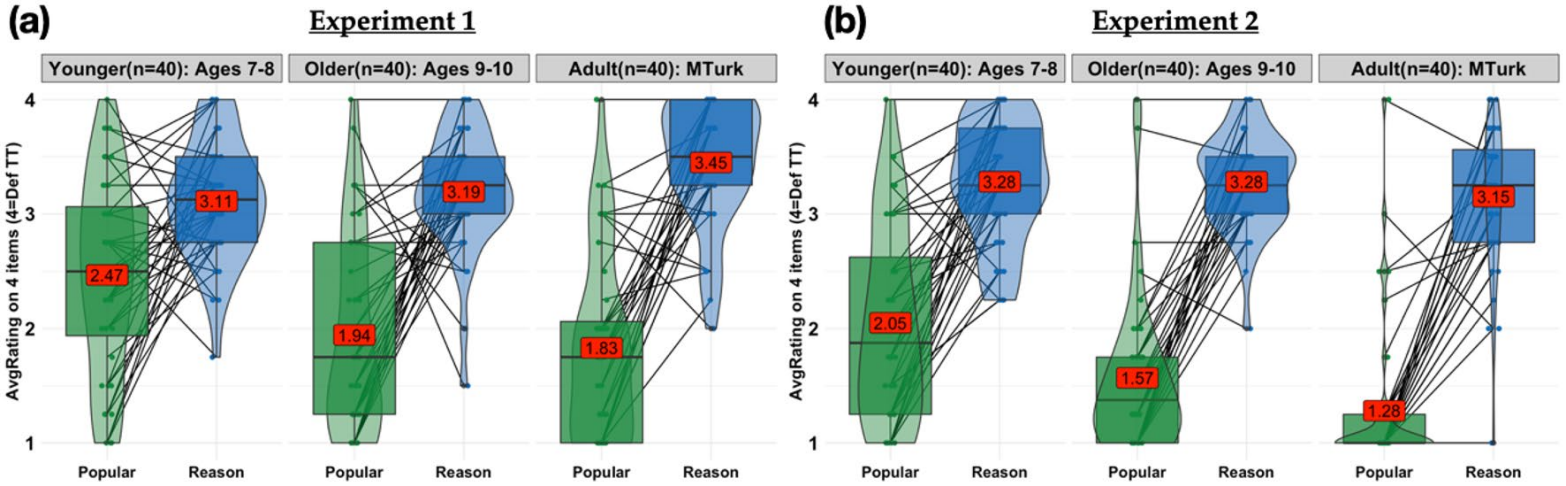
Experiments 1-2: Results. Preference for group discussion or independent crowd poll for (**a**) Experiment 1 (**b**) Experiment 2, averaged across four *Reasoning* questions (blue boxplots) and four *Population Preference* questions (green boxplots). Group discussion was 5 people in both experiments; crowd was 5 people in Experiment 1, and 50 people in Experiment 2. Higher ratings indicate stronger preference for group discussion. Boxplots showing median and interquartile range overlay violin plots; red labels show means; black lines show within-subject differences for the average rating by question *Type*.

 For the primary test, the four responses within each question domain (Fig. 2a) were averaged to create a single score for each domain. A repeated measures ANOVA revealed a significant effect of question *Type* (F(1,117) = 132.87, *p* < 0.001, η_*p*_^*2*^ = 0.532) and an AgeGroup*Type interaction (F(2,117) = 7.83, *p* < 0.001, η_*p*_^*2*^ = 0.118), and a marginal but non-significant effect of *AgeGroup* (F(2,117) = 2.82, *p* = 0.064, η_*p*_^*2*^ = 0.046). Multiple comparisons suggested that intuitions about how to manage collective wisdom appear by at least age 7: consistent with the empirical literature suggesting that group reasoning outperforms individual reasoning, all age groups believed that *Talking Together* would be more helpful than *Answering Alone* for *Reasoning* questions, both as compared to *Popularity* questions (Bonferroni corrected, Younger: *t*(117) = 3.66 *p* = 0.0057, Older: *t*(117) = 7.105, *p* < 0.0001, Adult: *t*(117) = 9.201, *p* < 0.0001), and compared to chance (Younger: M = 3.11, SD = 0.52, *t*(39) = 7.42, *p* < 0.0001, Older: M = 3.19, SD = 0.51, *t*(39) = 8.53, *p* < 0.0001, Adult: M = 3.45, SD = 0.54, *t*(39) = 11.237, *p* < 0.0001). Moreover, both Older children and Adults favored *Answering Alone* over *Talking Together* for *Popularity* questions, though Younger children’s answers for *Popularity* did not differ significantly from chance (Younger: M = 2.46, SD = 0.85 *t*(39) =  − 0.232, *p* = n.s., Older: M = 1.94, SD = 0.86, *t*(39) =  − 4.076, *p* < 0.001, Adult: M = 1.83, SD = 0.81, *t*(39) =  − 5.24, *p* < 0.0001). The preference for group reasoning did not differ by age (all *p*s > 0.4), though Older children and Adults showed a stronger preference for crowdsourcing *Popularity* questions than Younger children (Bonferroni corrected: Adult vs. Older: *t*(78) = 0.718, *p* = *ns*; Adult vs. Younger: *t*(78) = 4.067, *p* < 0.001; Older versus Younger: *t*(78) = 3.350, *p* < 0.0143). This developmental shift towards *Answering Alone* when discussion provides no objective criteria for evaluating accuracy is slightly earlier than we had predicted, but consistent with past work on children’s evaluation of non-independent testimony^26^.

Finally, all ages agreed that a teacher who wanted a group of five students to answer test questions accurately should have the students *Talk Together*, while a teacher who wanted to know which students had done their homework should have the students *Answer Alone* (Comp_AA: M_Young_ = 65%, *p* = 0.04, M_Old_ = 87.5%, *p* < 0.0001, M_Adult_ = 92.5%, *p* < 0.0001, Comp_TT: M_Young_ = 70%, *p* = 0.008, M_Old_ = 85%, *p* < 0.0001, M_Adult_ = 87.5%, *p* < 0.0001). This suggests that by age 7, children recognize that discussion could undermine inferences about individuals’ “independent” beliefs, but expect group discussion to either generate or disseminate accurate answers.

Taken together, these two tasks suggest that sophisticated intuitions about the risks and benefits of social influence may guide decisions about how to learn from collective judgment. Notably, these intuitions are consistent with empirical findings documenting the a group advantage over individuals for reasoning questions, and the value of independent responding when discussion is likely to bias collective judgment.

## Experiment 2

Could Experiment 1 have underestimated the value of crowdsourcing? Crowdsourcing may be most valuable with large crowds: larger crowds are more likely to include at least one accurate individual, and better represent the relative frequency of beliefs in the population. Moreover, in large enough crowds, even a minimal plurality will easily outnumber the unanimous consensus of a small group. Thus, if a belief’s frequency is a cue to its accuracy, a large crowd will always be more informative than a small group. In Experiment 2, we contrasted the 5-person group with a larger 50-person crowd. We predicted that since the *Popularity* questions simply ask the group or crowd to estimate what *most* people in a population prefer, all age groups would find it intuitive to ask *more* people—i.e., the crowd. The benefit of large crowds is less clear for *Reasoning* questions. If few individuals can solve a problem alone, identifying the correct answer in the crowd may be akin to finding a needle in a haystack; indeed, if individual accuracy is known to be rare, the most common answer may be a widely-shared misconception^53^. Yet, if many individuals can solve the problem alone, large crowds are redundant and a learner can outsource evaluating accuracy to a group discussion. We therefore predicted that adults and older children would continue to favor group deliberation over crowdsourcing for *Reasoning* questions. However, we saw two plausible alternatives for younger children. First, younger children could show the mature pattern. Alternatively, younger children’s preference for reasoning in groups could be attenuated by a “more is better” bias. Additionally, since the only difference between Experiments 1 and 2 was the increased crowd size, our design also allows us to explore the effects of crowd size itself by comparing the two experiments directly.

### Method

#### Participants

We recruited 40 adults through MTurk, as well as 80 children (40 Younger, M = 8.01, SD = 0.56; 40 Older, M = 9.92, SD = 0.56; 39 girls). As in Experiment 1, children participated through an online platform for developmental research that allows researchers to video chat with families using pictures and videos on slides^53^. One additional child was excluded and replaced because the family lost internet connection partway through the experiment and could not rejoin.

#### Materials and procedure

 The materials and procedure were identical to Experiment 1, but participants were first shown a large crowd of people, and told that Jack could either ask 5 of them to Talk Together, or 50 of them to Answer Alone. The answer choices from Experiment 1 were altered to display fifty cartoon icons for *Answering Alone* instead of five.

#### Results

 As before, the four responses for each question *Type* (Fig. 2b) were averaged to create a single score for each *Type.* A repeated measures ANOVA revealed a significant effect of *Type* (F(1,117) = 376.88, *p* < 0.001, η_*p*_^*2*^ = 0.763) and *AgeGroup* (F(2,117) = 9.63, *p* < 0.001 η_*p*_^*2*^ = 0.141), and an AgeGroup*Type interaction (F(2,117) = 5.39, *p* < 0.01, η_*p*_^*2*^ = 0.084). Despite the crowd having ten times as many sources as the group, participants were not swayed by a “more is better” bias; all age groups continued to prefer the group discussion for *Reasoning* questions, both as compared to *Popularity* questions (Bonferroni corrected, Younger: *t*(117) = 8.60 *p* < 0.0001, Older: *t*(117) = 11.97, *p* < 0.0001, Adult: *t*(117) = 13.06, *p* < 0.0001), and compared to chance responding (Younger: M = 3.28, SD = 0.53, *t*(39) = 9.29, *p* < 0.0001, Older: M = 3.28, SD = 0.42, *t*(39) = 11.75, *p* < 0.0001, Adult: M = 3.15, SD = 0.65, *t*(39) = 6.37, *p* < 0.0001). Moreover, even younger children in Experiment 2 favoured *Answering Alone* for *Popularity* questions, suggesting that they recognized that a large crowd would provide a better estimate of population preferences than a small group (Younger: M = 2.05, SD = 0.84, *t*(39) =  − 3.38, *p* = 0.0017, Older: M = 1.57, SD = 0.69, *t*(39) =  − 8.52, *p* < 0.0001, Adult: M = 1.28, SD = 0.66, *t*(39) =  − 11.75, *p* < 0.0001). As in Experiment 1, the preference for group reasoning did not differ by age (all *p*s > 0.9), though Older children and Adults again showed a stronger preference for crowdsourcing *Popularity* questions than Younger children (Bonferroni corrected, Adult versus Older: *t*(78) = 1.995, *p* = *ns*; Adult versus Younger: *t*(78) = 5.334, *p* < 0.001; Older vs. Younger: *t*(78) = 3.339, *p* < 0.0147). We also conducted two exploratory analyses of the effect of crowd size. Our preregistered prediction in Experiment 2 was that participants would favor the crowd for population preference questions, but continue to favor the group for reasoning questions. However, because the only difference between Experiments 1 and 2 was the increase in crowd size from 5 to 50 people, our data also enables us to test the crowd-size effect directly. We ran separate ANOVAs for each *QuestionType* using *AgeGroup* & *Experiment* as predictors. The tenfold increase in crowd size had no impact on participants’ preference for discussing *Reasoning* questions in small groups (F(1, 234) = 0.045, *p* = 0.8320); an AgeGroup*ExpNum interaction was significant (F(2,234) = 4.434, *p* = 0.0129), but post-hoc comparisons revealed only a marginal difference between younger children’s and adults’ preferences for reasoning in groups in Exp 1, with no other differences. However, participants were significantly more likely to crowdsource *Popularity* questions in Experiment 2 than Experiment 1 (F(1, 234) = 19.303, *p* < 0.0001), with no differences between age groups.

As in Experiment 1, responses to the comprehension questions at the end of the task suggested even the youngest children recognized that talking together would make it impossible for the teacher to know which students had done their homework (Comp_AA: M_Young_ = 67.5%, *p* = 0.019, M_Old_ = 92.5%, *p* < 0.0001, M_Adult_ = 90%, *p* < 0.0001). However, while older children and adults agreed that the students would do better on the test if they could discuss their answers, younger children were at chance (Comp_TT: M_Young_ = 52.5%, *p* = 0.4373, M_Old_ = 90%, *p* < 0.0001, M_Adult_ = 90%, *p* < 0.0001). Younger children may be less confident in the value of discussion than their responses to the main task questions in Experiments 1 and 2 would suggest; however, informal questioning of participants after the experiment suggested that younger children in Exp 2 may have simply rejected talking together on a test as cheating, even though the question specified that the teacher could choose to allow students to talk together.

In short, Experiment 2 suggests that young children’s intuitions about the value of group discussion are consistent with empirical demonstrations of a group advantage for reasoning questions and the value of large crowds for intuitive estimations. Moreover, directly comparing Experiments 1 and 2 suggests that while children’s preference for reasoning in small groups is stable even in the face of a much larger crowd, they also recognize that for some questions, larger crowds are more helpful than smaller crowds.

## Experiment 3

Using *Population Preferences* as the Non-Reasoning questions in Experiments 1 and 2 leaves two points unclear. First, since a culture’s preferences are intuitive for most people, the *Popularity* questions may have simply seemed easier to answer than the *Reasoning* questions. Second, because individual preferences are literally constitutive of the population preference, children’s responses could reflect an understanding of the nature of preference polling as much as an understanding of the potential for groupthink. To test these two alternatives, we contrasted easy versions of the reasoning questions with challenging perceptual discrimination questions. Disagreement about a challenging perceptual discrimination task would leave a group of laypeople little to discuss beyond confidence, which may be sufficient to filter out obviously wrong answers^56,57^, but is generally an unreliable proxy for accuracy. In contrast, polling a large crowd has been shown to increase the accuracy of a collective decision for perceptual tasks^58^. The relative difficulty of the *Easy Reasoning* and *Hard Percept* items was confirmed in a pre-test (Supplemental Materials). If participants’ preference for group discussion in Experiments 1 and 2 was driven by perceived question difficulty, then they will prefer group discussion for *Hard Percept* questions more than for *Easy Reasoning* questions. If group discussion was preferred because of its perceived benefits for reasoning, participants will prefer group discussion more for *Easy Reasoning* than *Hard Percept*. If participants recognize the risks of social influence when discussants cannot rely on demonstrative reasoning, they will prefer crowdsourcing for *Hard Percept* questions. We predicted that adults and older children would recognize the tradeoffs, but because children under 8 frequently fail to recognize the potential for motivational biases even in simpler cases^59^, we predicted that younger children would prefer group discussion for both question types. As an exploratory analysis, we also compare the results in Experiment 3 directly to Experiment 2, but given that both the perceived difficulty and the subtype of Non-Reasoning question differ between Experiments, direct comparisons should be interpreted with caution.

### Method

#### Participants

 We recruited 40 adults through MTurk, as well as 80 children (40 Younger, M = 8.01, SD = 0.62; 40 Older, M = 10.00, SD = 0.53; 37 girls). As in Experiments 1 & 2, children participated through our online platform^54^. Two children were excluded and replaced when database records identified them afterwards as having already participated in Experiment 2. Two adults were excluded and replaced as well; though our preregistered plan was to accept all MTurkers who passed the basic attention screening, two worker identification codes appeared multiple times in the data, passing the attention screen after failing and being screened out two and three times, respectively, in violation of MTurk policies.

#### Materials and Procedure

 Methods were identical to Experiment 2, with the exception of the following changes made to the questions themselves. First, we presented four new Non-Reasoning questions, replacing the four *Popularity* questions with four *Percept* questions: (1) decide which of two pictures of a face “at the tipping point of animacy” is a photo and which is a photorealistic drawing^60^, (2) decide whether an opaque box contains 30 or 40 marbles by listening to a recording of it being shaken^61^, (3) identify which of twelve colored squares in a visual array is rotating the fastest, and (4) rank the 25 brightest stars in a photo of the night sky in order of brightness. Second, we simplified the four *Reasoning* questions (see Supplemental Materials) by (1) completing most of the Sudoku, (2) reducing the number of treasures Mario was required to pick up in the vehicle routing problem, and (3) replacing the “impossible object bottle” with an analog of the “floating peanut” task, which requires the learner to extract an object from a jar of water without touching the jar or object^62^. The fourth Reasoning question, Nim, remained the same, as adults rated the 5-item Nim heap as easy to solve.

#### Results

**Figure 3:**
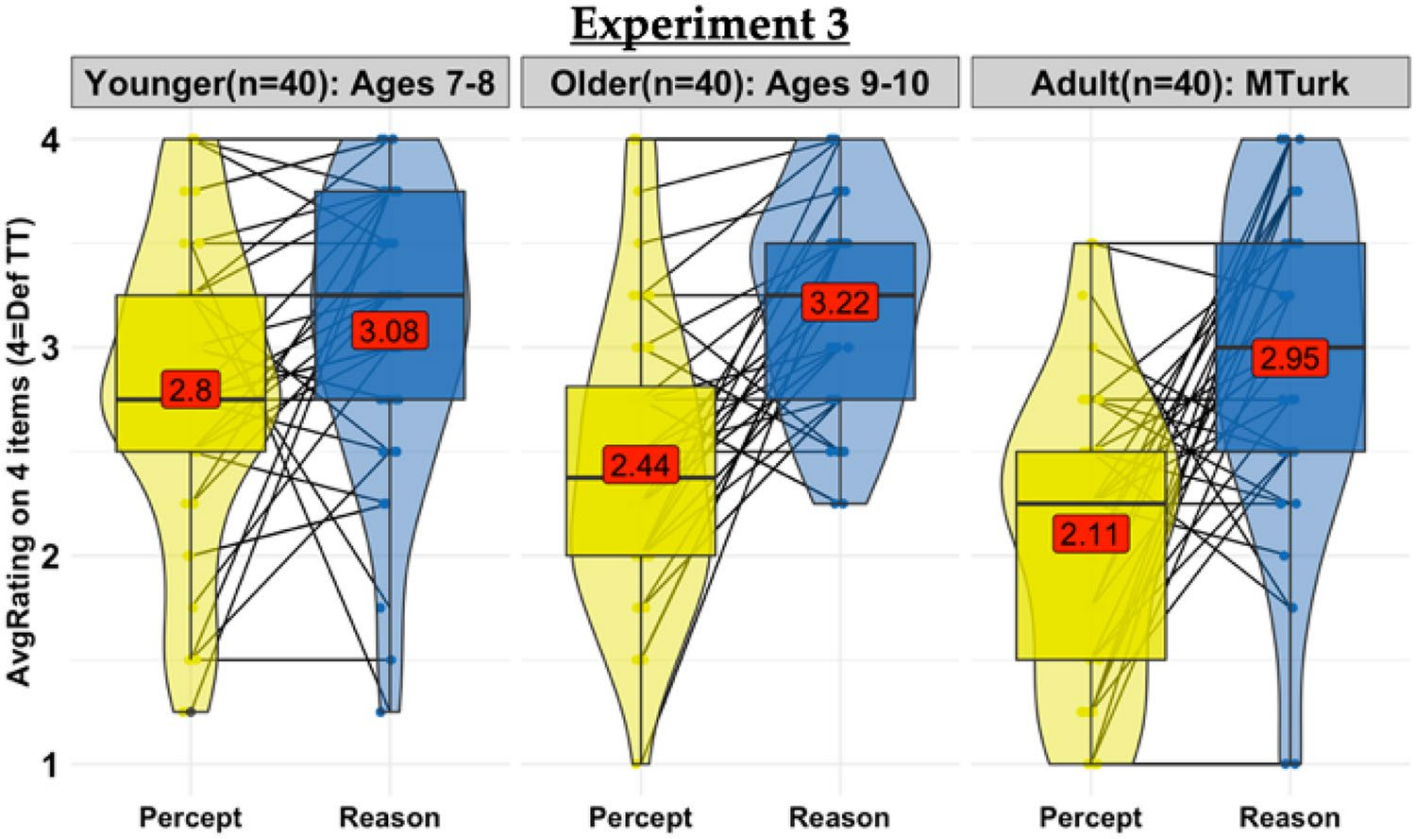
Experiment 3: Results. Preference for 5-person group discussion or independent crowd poll of 50 people, averaged across four *Easy Reasoning* questions (blue boxplots) and four *Hard Perceptual Discrimination* questions (yellow boxplots). Higher ratings indicate stronger preference for group discussion. Boxplots showing median and interquartile range overlay violin plots; red labels show means; black lines show within-subject differences for the average rating by question *Type*.

 For the primary test, the four responses within each question domain (Fig. 3) were again averaged to create a single score for each *Type*. A repeated measures ANOVA again revealed a significant effect of question *Type* (F(1,117) = 56.12, *p* < 0.0001, η_*p*_^*2*^ = *0.324*) and *AgeGroup* (F(1,117) = 7.01, *p* = *0.0*012, η_*p*_^*2*^ = *0.108*) an AgeGroup*Type interaction (F(2,117) = 4.40, *p* = 0.0143, η_*p*_^*2*^ = *0.070*). The perceived difficulty of the questions had no discernible effect on participant judgments: participants of all ages again rejected the large crowd in favor of the small group discussion for the *Easy Reasoning* questions (Younger: M = 3.08, SD = 0.69 *t*(39) = 5.35, *p* < 0.0001, Older: M = 3.22, SD = 0.51, *t*(39) = 8.97, *p* < 0.0001, Adult: M = 2.95, SD = 0.78, *t*(39) = 3.64, *p* = 0.0008). We also observed the predicted developmental shift towards *Answering Alone* when reasoning was insufficient to answer the question; however, in Experiment 3 the shift occurred later than expected instead of earlier. While Adults favored *Answering Alone* and Younger children favored *Talking Together* for the *Hard Percept* questions as predicted, Older children did not show the adult pattern, instead not differing from chance for *Hard Percept* (Younger: M = 2.80, SD = 0.71, *t*(117) = 2.66 *p* = 0.011; Older: M = 2.44, SD = 0.71, *t*(117) = -0.55, *p* = 0.7535; Adult: M = 2.11, SD = 0.69, *t*(117) =  − 3.62, *p* = 0.0008). Moreover, while Older children and Adults distinguished between the two question *Types*, Younger children did not (Bonferroni corrected, Younger: *t*(117) =  − 1.91 *p* = 0.8701, Older: *t*(117) =  − 5.318, *p* < 0.0001, Adult: *t*(117) = 5.74, *p* = 0.0001). As in Experiments 1 and 2, the preference for group reasoning did not differ by age (all *p*s > 0.9), though Younger children showed a weaker preference than Adults for crowdsourcing *Percept* questions (Bonferroni corrected, Adult vs. Older: *t*(78) = 2.156, *p* = *ns*; Adult vs. Younger: *t*(78) = 4.516, *p* < 0.0002; Older vs. Younger: *t*(78) = 2.360, *p* = *ns*). Indeed, while participants of all ages were just as confident in the small group discussion for *Easy Reasoning* questions in Experiment 3 as they were for *Reasoning* questions in Experiment 2, all ages were less confident in polling a crowd of 50 for *Hard Percept* questions in Experiment 3 than for *Population Preferences* in Experiment 2 (Supplemental Materials). However, since Experiment 3 was designed contrast *Easy Reasoning* questions with *Hard Percept* questions, rather than *Hard Percept* with *Population Preferences*, these direct comparisons with Experiment 2 should be interpreted with caution: for example, the weaker preference for crowdsourcing *Hard Percept* questions than *Population Preference* questions may be due the difference in Non-Reasoning subtype or an effect of difficulty that is specific to Non-Reasoning questions. We explore these possibilities further in the General Discussion.

## General discussion

We asked children and adults to choose between two social learning strategies: soliciting a consensus response from a small discussion group, and “crowdsourcing” many independent opinions. Though discussion can sometimes lead to groupthink, by affording individuals opportunity to correct each others’ mistakes and combine insights while also reducing individual processing load, discussion can also allow small groups to outperform even their best member. In contrast, the value of crowdsourcing is fundamentally limited by the distribution of individual competence in the crowd relative to its size. The less competent individuals are on average, the larger the crowd needs to be to produce a reliably accurate estimate. Thus, when individual competence is low, crowdsourcing may be costly; when individual competence is high, the value added by crowdsourcing may have little advantage over discussion—for problems where discussion is more likely to improve accuracy than diminish it. Our results suggest that the decision to crowdsource or discuss may in part turn on learners’ beliefs about the efficacy of demonstrative reasoning for a given question.

Analogously to young children’s failures on false belief tasks, our results suggest that the default expectation for group judgments may be that “truth wins”: though individuals may initially disagree, discussion allows groups to ultimately see the truth. As an understanding of how conscious and unconscious biases can influence people’s judgments develops, learners can preempt potential biases by crowdsourcing independent judgments. Though even the youngest children in our experiments expected discussion to improve accuracy on reasoning questions, the preference for crowdsourcing non-reasoning questions underwent a developmental shift in all three experiments. Indeed, in Experiment 3, the youngest children favored discussion for both kinds of questions, suggesting that they may have failed to recognize when discussion can promote groupthink. The timing of the developmental shift is consistent with past work suggesting that between the ages of 6 and 9, children begin to use informational dependencies^21–26^ and the potential for motivational bias in individual reports^29,59^ to adjudicate cases of conflicting testimony. Though recent work suggests that even preschoolers identify cases of individual bias stemming from in-group favoritism^63^, unconscious biases due to herding or groupthink may be less obvious, particularly if people assume that informants are motivated to be accurate. For example, even though children as young as six *predict* that judges are more likely to independently give the same verdict when objective standards are available than when they are not (e.g., a footrace vs. a poetry contest), at age ten children are still no more likely to *diagnose* in-group favoritism as an influence on judgments in subjective contexts than objective contexts^28,63^. In our experiments, both the reasoning and non-reasoning questions had objective answers, but only the reasoning questions afforded an objective *method of finding* those answers. Learning to recognize this relatively subtle distinction may allow children to take advantage of the benefits of group discussion while avoiding the risks. This is not to suggest that people expect group reasoning to be infallible—merely that they expect groups to improve individual accuracy. This is consistent with recent work asking adults to predict group and individual accuracy on a classic reasoning task: while participants radically underestimated the true group advantage, they did expect groups to be more accurate than individuals^64^. Interestingly, they also expected *dyads* to be *less* accurate than individuals. A more granular approach to intuitive beliefs about the dynamics of social influence may reveal more sophisticated intuitions: for instance, beliefs about others’ conformist tendencies and the distribution of individual competence may increase confidence that “truth wins” in small groups more than in dyads.

Past work has suggested that while people dramatically underestimate crowds and overestimate their own accuracy^17,65^, they defer to others more when uncertainty is high and crowds are larger^66,67^. While increasing the crowd size from five to fifty had no impact on *Reasoning* questions in our experiments, the larger crowd did appear to increase crowdsourcing for *Population Preference* questions. However, while we only tested *Hard Percept* questions with a crowd of fifty, confidence in crowdsourcing was lower for *Hard Percept* questions than for *Population Preferences* (Supplemental Materials). While our design licenses no firm conclusions on this point, one reason seems evident: by definition, population preferences are whatever most individuals in a population prefer, while perceptual facts like the brightness of stars are wholly independent of individual judgments. Moreover, under the right conditions, discussing perceptual judgments with a single partner *can* improve accuracy^68,69^. Thus, participants’ reduced confidence in crowdsourcing *Hard Percept* questions may have been justified. The extent to which intuitive beliefs about the benefits of discussion and crowdsourcing for different question types correspond to the empirical benefits is an open question.

Our design is limited in one important respect: the discussion group was only allowed to give a single answer, while the crowd could give multiple answers. This procedure strictly ensured that group members could not answer independently, but also entailed a unanimous consensus endorsed by a minimum of five people. Unanimous consensus can be a powerful cue: even a single dissenter can sharply reduce conformity^24,67^. However, the meaning of dissent may vary across contexts and questions. In a crowd, a single “dissenter” may simply have made a mistake; but dissent-despite-discussion signals that the group has failed to convince them. When questions afford conclusive demonstrations of accuracy, failure to convince all discussants may reflect poorly on group accuracy. Conversely, in more ambiguous contexts, unanimity may suggest groupthink. For instance, in ancient Judea, crimes more likely to elicit widespread condemnation were tried by larger juries for the express purpose of reducing the odds of consensus, and unanimous convictions were thrown out on the grounds that a lack of dissent indicated a faulty process—an intuitive inference confirmed by modern statistical techniques^70^. A similar logic may underlie inferences about testimony that contradicts social alliances. For example, if Jenny says Jill is bad at soccer, even preschoolers give Jenny’s judgment more credence if Jenny and Jill are friends than if they are enemies^63^. Our results suggest that even in early childhood, the absolute number of sources endorsing a belief may be less important than how those sources arrived at their beliefs. Indeed, the limited number of possible answers to the questions in Experiments 2 and 3 guaranteed that even a plurality of the 50-person crowd would considerably outnumber the 5-person group. Yet, participants’ preference for discussion and crowdsourcing bore no relationship to the number of possible endorsers. Future work will compare explicit degrees of consensus in groups and crowds.

The last decade has produced an extensive literature describing how individual social learning heuristics and patterns of communication in social networks can improve or diminish collective learning^33,71–73^. By focusing on population-level outcomes, much of this work has tacitly treated individuals as passive prisoners of social influence. However, the heuristics guiding social learning develop in early childhood, and recent work has shown that like other intelligent systems capable of self-organization, people are capable of “rewiring” their social networks to improve both individual and collective learning, by “following” or “unfollowing” connections depending on their accuracy^73^. Our experiments focused on two features of communication patterns that individuals can and do control in the real world, beyond *who* they choose to trust: how many people to talk to, and whether to talk with those people as a group or a crowd. Our results suggest that even in early childhood, people’s judgments about how to best make use of group discussion and crowdsourcing heuristics may be consistent with the empirical advantages of each strategy. An understanding of how intuitions about social influence develop may contribute to a clearer empirical picture of how people balance the benefits of learning from collective opinion with the risks of being misled by it.

## Supplementary Information

Supplementary Information.
