# Supplementary material for: The potential for effective reasoning guides children’s preference for small group discussion over crowdsourcing
Source: Sci Rep. 2022 Jan 24;12:1193. doi: 10.1038/s41598-021-04680-z (PMC8786842; doi:10.1038/s41598-021-04680-z)
Supplement: Supplementary file 1 — Supplementary Information. [file 41598_2021_4680_MOESM1_ESM.docx]

**Supplemental Materials**

**I. Experiments 1 & 2: Comprehension Questions**

After the test questions in Experiments 1 and 2, we asked two comprehension questions (“Comp_TT” and “Comp_AA”) to test more explicitly whether participants were considering the effects of information sharing in a setting familiar to children. In these questions, Jack’s teacher was giving a test to Jack’s 5 informants, and participants were asked whether the 5 people should answer by Talking Together or by Answering Alone. In Comp_TT, the teacher wanted “the 5 people to get as many answers right as possible”; in Comp_AA, the teacher wanted to “find out which of the 5 people did their homework and which ones didn’t”. If children understand how discussion changes the informativeness of individual responses, they should recognize that Answering Alone is more informative to the teacher in Comp_AA. If they understand the benefits of discussion (or at least, information sharing), they should prefer Talking Together for Comp_TT.

In Experiment 1, children’s responses to the comprehension questions suggest that even the youngest were able to choose a method of responding consistent with what the teacher wanted to learn about the students (Comp_AA: M_Young_= 65%, *p*=.04, M_Old_= 87.5%, *p*<.0001, M_Adult_= 92.5%, *p*<.0001, Comp_TT: M_Young_= 70%, *p*=.008, M_Old_= 85%, *p*<.0001, M_Adult_= 87.5%, *p*<.0001).

As in Experiment 1, responses to the comprehension questions at the end of Experiment 2 suggested even the youngest children recognized that talking together would make it impossible for the teacher to know who had done their homework (Comp_AA: M_Young_= 67.5%, *p*=.019, M_Old_= 92.5%, *p*<.0001, M_Adult_= 90%, *p*<.0001). However, while older children and adults recognized that the students would do better on the test if they could discuss their answers, younger children were at chance (Comp_TT: M_Young_= 52.5%, *p*=.4373, M_Old_= 90%, *p*<.0001, M_Adult_= 90%, *p*<.0001). Children in Experiment 2 may have been less confident in the value of discussion than their responses to the the main task questions in Experiments 1 and 2 would suggest; however, informal questioning of participants after the experiment suggested that younger children in Exp 2 may have simply rejected talking together on a test as cheating, even though the question specified that the teacher themselves could choose to allow students to talk together.

**II. Supplementary Methods for Experiment 3**

**Norming Experiment.** In order to confirm the difficulty level of the Hard Percept and Easy Reasoning questions in Experiment 3, we first ran a norming experiment on MTurk with a separate group of 42 adult participants. Three participants were screened out for failing to answer basic comprehension questions about their job in the HIT.

We created 8 questions (4 Percept and 4 Reasoning) that we expected participants to rate as “easy” to answer and another 8 questions (4 Percept and 4 Reasoning) that we expected participants to rate as “hard” to answer. Each participant saw 8 questions: either the 4 Easy Reasoning and 4 Easy Percept questions, or the 4 Hard Reasoning and 4 Hard Percept questions. We expected the Hard Percept questions to be rated as more difficult to answer correctly than the Easy Reasoning questions. Each participant was asked “How difficult would it be to answer the question?”, and rated the difficulty on a 7 point scale, from *Extremely easy* to *Extremely difficult*.

The *Percept* questions:
**Photorealism:** decide which of two pictures of a face is a photo and which is a photorealistic drawing made by a talented artist. These materials adapted from Looser & Wheatley, 2010, which morphed faces using photographs and dolls as the anchors. We used Morph 3. The Easy version used Morph3_052Human and Morph3_067Human. The Hard version used Morph3_063Human and Morph3_065Human.
**Intuitive Psychophysics (Superballs):** decide how many marbles an opaque box contains by listening to it being shaken. This task was adapted from Siegel, Magid, Tenenbaum & Schulz, 2014. Two recordings were created. The Easy version asked whether the box contained 2 or 10 marbles (the recorded version contained 2). The Hard version asked whether the box contained 30 or 40 marbles (the recorded version contained 40).

Brightness (Stars): decide which of the stars in a starry night sky looked the brightest. A picture of a starry night sky over a desert was used to represent the night sky, and the protagonist was said to have taken the picture so that he could “circle the brightest ones”. In the Easy version, he wanted to circle the 3 brightest stars. The Hard version he wanted to circle the 25 brightest stars.

**Rotation Speed**: identify which of twelve colored diamonds is rotating the fastest. Each diamond had an A, a K, or a W in it to make the rotation clearer, but in the Hard version, the diamonds all had approximately the same RPM, while in the Easy version, the RPM was overall slower, and one was a clear outlier. The matrixes below show the number of rotations of each item in the Hard and Easy 4x3 arrays during the 10s display. In the Hard array, the fastest made 27 rotations in 10s, but 3 others made 26 and 2 made 25 rotations. In the Easy array, the fastest made 19 rotations in 10s, and the next closest made 12.

| Hard (# Rotations/10s) | Easy (# Rotations/10s) |
| --- | --- |
| 24 22 21 **27**  26 26 26 25  25 22 25 22 | 8 6 10 7  6 3 7 **19**  8 9 12 11 |

The *Reasoning* questions: The reasoning questions were adapted from Experiments 1 and 2.
**Sudoku**: Experiments 1 and 2 used a 4x4 sudoku problem rated as “easy” in a compilation, replacing the numbers with fruit to make it kid-friendly. The Easy version in Experiment 3 completed two additional moves. The Hard version used a 9x9 rated as “hard” in a compilation.

**Vehicle Routing Problem:** Experiments 1 and 2 used a custom made pathfinding puzzle which required a MarioKart find the shortest road through all the treasures on a map without taking “two in a row that are the same color, or two in a row that are the same shape”. The Hard version used in these experiments had 11 treasures of different shapes and colors scattered randomly around the map. The Easy version created for Experiment 3 reduced the number of treasures to 4, of only 3 shapes and colors.
**Bottle-Jar Extraction Task**: Experiments 1 and 2 presented an “impossible object” puzzle, requiring the solver to remove a stick from a bottle without breaking the bottle or the stick. The stick was held fast inside the bottle by a nut-and-bolt. This was used as the Hard version. The Easy version substituted an analog of the “floating peanut” task (e.g., (Hanus, Mendes, Tennie, & Call, 2011), requiring the solver to remove a rubber ducky from large open-neck jar half-full of water, without touching the ducky or the jar, by pouring in the water from another jar.

**Nim**: In the game of Nim, each side takes turns picking up pencils. Each turn, you have to pick up either one, two, or three pencils. The winner is the person who picks up the last pencil. In Experiments 1 and 2, the we showed a game with only 5 pencils left. As adults and some older children found this 5-item version easy to solve, we created a Hard version by leave 22 pencils, and emphasizing that a wrong move would let a “super-smart computer” opponent win.

**Norming Experiment**: **RESULTS**. We fit a mixed effects model to perceived difficulty ratings, with random slopes and intercepts for each participant and question to account for repeated measures. The model confirmed that participants expected the Hard questions to be more difficult to answer than the Easy questions, (β = 1.95, SE = .5523, *p* = .0055). With the exception of the Easy version of the Percept_Stars question, which was rated as significantly more difficult than other Easy questions (β = 2.45, SE = .0.4988, *p* < .0001), the questions within each difficulty level did not differ amongst themselves in perceived difficulty. Experiment 3 contrasted the Easy versions of the Reasoning questions with the Hard versions of the Percept questions; if participants preference for group reasoning in Experiments 2 and 3 was driven by the perceived difficulty of the question, then participants in Experiment 3 will favor group reasoning more for the *Hard Percept* questions than the *Easy Reasoning* questions.

**III. Cross-Experiment Exploratory Analyses**

We conducted several exploratory analyses comparing results between experiments to examine the effects of crowd size and and question type more broadly. Experiment 1 and Experiment 2 used identical questions, but Experiment 2 increased the size of the crowd from 5 to 50 people. Our preregistered prediction was that participants would favor the crowd for population preference questions, but continue to favor the group for reasoning questions. However, we can also test the direct effect of crowd size by comparing people’s judgments for reasoning and for popularity questions in Experiment 1 to their judgments in Experiment 2. Experiment 3 again used a crowd of 50 people, but contrasted easy versions of the reasoning questions from Experiments 1 and 2 with challenging perceptual discrimination tasks. This allowed us to test whether the preference for group discussion was caused by the perceived difficulty of the question. However, it also allows us to test whether the preference for crowdsourcing observed in Experiments 1 and 2 extended to questions with a more ambiguous relationship to crowd size than population preferences.

To explore the effect of crowd size, we ran separate ANOVAs for each QuestionType using AgeGroup & Experiment as predictors (Exps 1 and 2). The tenfold increase in crowd size had no impact on participants’ preference for discussing reasoning questions in small groups (F(1, 234)=0.045, *p=*.8320); an *AgeGroup*ExpNum* interaction was significant (F(2,234)=4.434, *p=*.0129), but post-hoc comparisons revealed only a marginal difference between younger children’s and adults’ preference for reasoning in groups in Exp 1, but no other differences. However, participants were significantly more likely to crowdsource popularity questions in Experiment 2 than Experiment 1 (F(1, 234)=19.303, *p<*0.0001), with no differences between age groups.

To explore whether the crowdsourcing preference was as strong for perceptual discrimination problems as population preference questions, we ran an ANOVA comparing the two types of non-reasoning questions, using AgeGroup & Experiment as predictors (Exps 2 and 3). Participants were significantly less confident that crowdsourcing would be preferable to a small group discussion for percept questions than popularity questions (F(1, 234)=76.897, *p* < 0.0001); the interaction was not significant (F(2,234)=0.139, *p=*.87). Notably however, there was no difference between participants’ preference for asking a small group to discuss *Easy Reasoning* questions in Experiment 3 and *Reasoning* questions in Experiment 2, though it did approach significance (F(1, 234)=3.858, *p* < 0.0507).

**IV. MTurk Quality Screen**

We present instructions as voice-over videos in order to prevent language bots from skimming the written text, and immediately after the videos, we simply present participants with 3 multiple choice questions about their task (*A: is their job to answer the questions themselves or decide which answer will help Jack more, B: do the people who answer alone talk together before each telling Jack their answer or not talk together, C: do the people who talk together each tell Jack their own answer after talking, or do they have to agree on a single answer to tell Jack after talking*), with the correct answer being a nearly verbatim transcript from the video. Participants who get 1 or more of the attention check questions wrong have one more opportunity to answer after watching the video again; if they get any questions wrong in the second round, they’re blocked from taking the survey.

**V. Supplemental Plot for Exps 1-3**

| 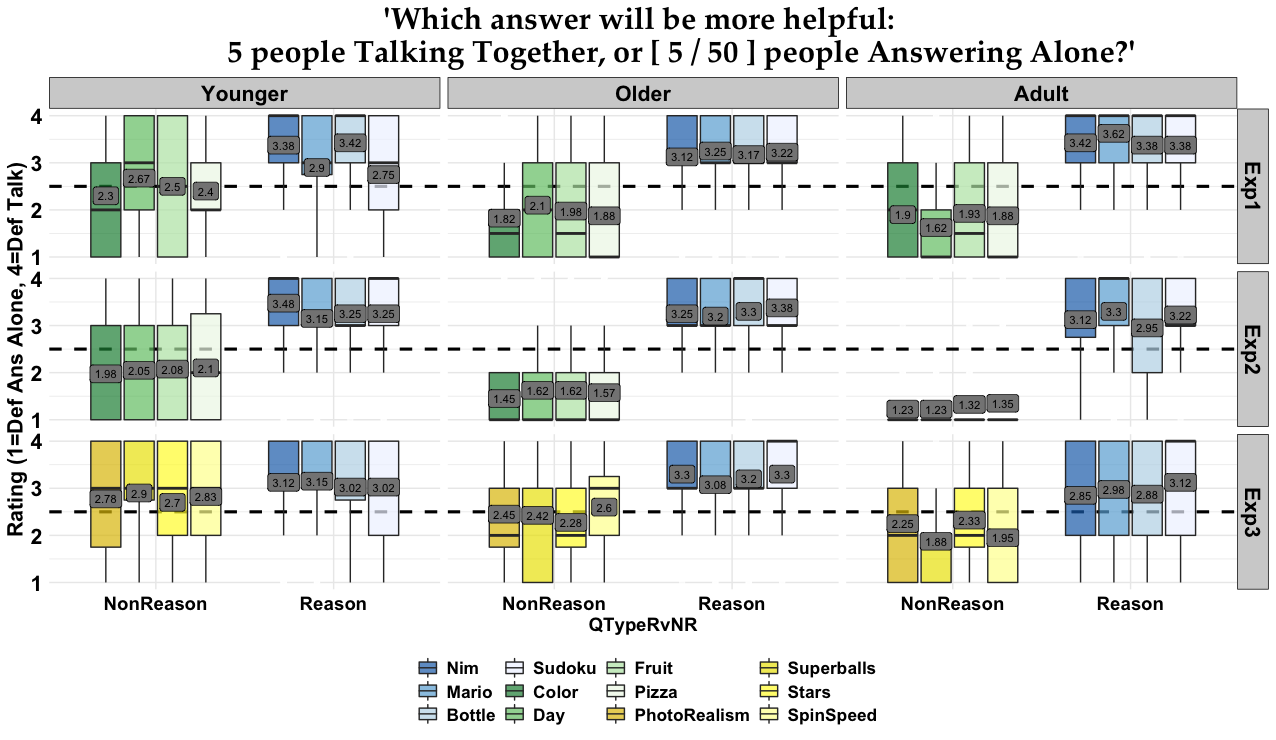 |
| --- |
| By-question boxplots for each of the 4 Reasoning questions (Blues) and 4 Non-Reasoning questions (Popularity - Greens; Percept - Yellows) in each experiment. Grey labels are means. (For preregistered analyses, average scores were computed for each QuestionType). |

**VI. Mixed Effects Models**

Our preregistered analysis plan was to compute an average score from the four questions of each QuestionType and conduct a repeated measure ANOVA on these two average scores. However, we also report mixed effects models; by including the un-averaged ratings for each question (i.e., the ratings on the 4-point scale for each of the four questions of each question type), these account for variance in the questions themselves. For each experiment, we tested the model (Ct_Rating ~ 0+AgeGroup*QuestionType + (1|subID), which models the responses for each of the 8 questions while treating AgeGroup and QuestionType as fixed effects, and allowing random intercepts for each subject. Centering individual ratings on 2.5 and deleting the intercept compares simple effect estimates to “chance” (i.e., 2.5 on a scale of 1 to 4) for each age group and estimates of interactions to the prior level’s interaction, testing our predictions versus chance for the reference level of QuestionType and versus the magnitude of the previous age group’s interaction for each interaction term; we report models with both Reasoning and Non-Reasoning questions coded as the reference level. These MEMs of raw ratings for each question produced qualitatively identical results to the repeated measures ANOVA on the averaged question ratings, with one exception: in Experiment 3, the mixed effect model suggested that while the youngest children favored group discussion for Non-Reasoning questions as well as Reasoning questions (consistent with the ANOVA), they also distinguished between the two (contrary to the ANOVA, where the difference was not significant), favoring discussion for Reasoning question more than for Non-Reasoning questions

**(A) Exp 1:** All age groups favored group discussion for Reasoning questions (β_Younger_ = .6125, *SE* = .086, *p* = 9.33e-12; β_Older_ = .69375, *SE* = .086, *p* = 2.15e-14; β_Adult_ = .950, *SE* = .086, *p* < 2e-16), as well as making increasingly stronger distinctions between Reasoning and Non-Reasoning questions with age (β_Younger_ = -.64375, *SE* = .10774, *p* = 3.40e-09; β_Older_ = -.60625, *SE* = .15236, *p* = 7.52e-05; β_Adult_ = .950, *SE* = .15236, *p* < 2.60e-10). Rerunning the regression with Non-Reasoning as the reference level showed that while younger children did not favor crowdsourcing for Non-Reasoning questions, older children and adults did (β_Younger_ = -.03125, *SE* = .086, *p* = 0.717; β_Older_ = -.55625, *SE* = .086, *p* = 4.62e-10; β_Adult_ = -.66875, *SE* = .086, *p* < 1.47e-13)

**(B) Exp 2:** As in Exp 1, all age groups favored group discussion for Reasoning questions (β_Younger_ = .78125, *SE* = .086, *p* < 2e-16; β_Older_ = .78125, *SE* = .086, *z* *p* < 2e-16; β_Adult_ = .65, *SE* = .086, *p* = 9.77e-13), as well as making increasingly stronger distinctions between Reasoning and Non-Reasoning questions with age (β_Younger_ = -1.23125, *SE* = .092, *p* < 2e-16; β_Older_ = -.48125, *SE* = .130, *p* = 0.000232; β_Adult_ = -.63750, *SE* = .130, *p* =1.16e-06). Rerunning the regression with Non-Reasoning as the reference level showed that all age groups favored crowdsourcing for Non-Reasoning questions (β_Younger_ = -.450, *SE* = .086, *p* = 3.73e-07; β_Older_ = -.93125, *SE* = .086, *p* < 2e-160; β_Adult_ = -.1.21875, *SE* = .086, *p* < 2e-16).

**(C) Exp 3:** All age groups favored group discussion for Reasoning questions (β_Younger_ = .58125, *SE* = .096, *p* = 5.64e-09; β_Older_ = .71875, *SE* = .096, *p* = 1.45e-12; β_Adult_ = .45625, *SE* = .096, *p* = 3.56e-06), as well as making increasingly stronger distinctions between Reasoning and Non-Reasoning questions with age (β_Younger_ = -.28125, *SE* = .106, *p* =0.008342; β_Older_ = -.500, *SE* = .150, *p* = 0.000926; β_Adult_ = -.575, *SE* = .150, *p =* 0.000142). Rerunning the regression with Non-Reasoning as the reference level showed that while younger children preferred to discuss Non-Reasoning questions as well, older children had no preference, and adults preferred crowdsourcing Non-Reasoning questions (β_Younger_ = .300, *SE* = .096, *p* = 0.002019; β_Older_ = -.06250, *SE* = .096, *p* = 0.516040; β_Adult_ = -.400, *SE* = .096, *p* = 4.4e-05).

More complex random effects specification were overfit or failed to converge, but suggested little variance between questions themselves after accounting for the effect of QuestionType. For instance, Model 1 (below) allows for random intercepts of questions within the fixed effect of QuestionType, but fit was singular. Inspecting random effects suggested that the (1|QuestionType:Question) explained no variance.

Model 1:

Ct_Rating ~ 0+QuestionType*AgeGroup+(1|subID)+(1|QuestionType:Question)
